# The wisdom of mistrust: qualitative insights from transgender women who participated in PrEP research in Lima, Peru

**DOI:** 10.1002/jia2.25769

**Published:** 2021-09-27

**Authors:** Amaya Perez‐Brumer, Sarah Naz‐McLean, Leyla Huerta, Ximena Salazar, Javier R Lama, Jorge Sanchez, Alfonso Silva‐Santisteban, Sari L Reisner, Kenneth H Mayer, Jesse L Clark

**Affiliations:** ^1^ Division of Social and Behavioural Health Dalla Lana School of Public Health University of Toronto Toronto ON Canada; ^2^ Department of Medicine Division of Infectious Diseases Brigham and Women’s Hospital Boston MA USA; ^3^ Feminas Lima Peru; ^4^ Universidad Peruana Cayetano Heredia Lima Peru; ^5^ Asociación Civil Impacta Salud y Educación Lima Peru; ^6^ Department of Global Health University of Washington Seattle WA USA; ^7^ Centro de Investigaciones Tecnologicas y Biomedicas Universidad Nacional de San Marcos Lima Peru; ^8^ Harvard T.H. Chan School of Public Health Boston MA USA; ^9^ The Fenway Institute Fenway Health Boston MA USA; ^10^ Beth Israel Deaconess Medical Center Boston MA USA; ^11^ Harvard Medical School Boston MA USA; ^12^ Department of Medicine Division of Infectious Diseases University of California Los Angeles David Geffen School of Medicine Los Angeles CA USA

**Keywords:** mistrust, PrEP, transgender, critical global health, HIV prevention

## Abstract

**Introduction:**

Although pre‐exposure prophylaxis (PrEP) is a remarkable biomedical advance to prevent HIV, ongoing research on PrEP contributes to and interacts with a legacy of HIV experimentation on marginalized communities in resource‐limited settings. This paper explores the complexity of PrEP research mistrust among Peruvian transgender (trans) women who completed a PrEP adherence intervention and those who refused participation (i.e. declined to enrol, voluntarily withdrew, and/or were lost to follow‐up).

**Methods:**

Data were derived from 86 trans women (mean age 29 years) participants in the formative (four focus groups (n = 32), 20 interviews) and the evaluation stages (34 interviews) of a social network‐based PrEP intervention for trans women in Lima, Peru. The formative stage took place from May to July 2015, while the evaluative stage took place from April to May 2018. Audio files were transcribed verbatim and analysed via an immersion crystallization approach using Dedoose (v.6.1.18).

**Results:**

Three paradoxes of trans women’s participation in PrEP science as a “key” population emerged as amplifying mistrust: (1) increases in PrEP research targeting trans women but limited perceived improvements in HIV outcomes; (2) routine dismissal by research physicians and staff of PrEP‐related side effects and the social realities of taking PrEP, resulting in questions about who PrEP research is really for and (3) persistent limitations on PrEP access for trans women despite increasing involvement in clinical trials, fostering feelings of being a “guinea pig” to advance PrEP science.

**Conclusions:**

Findings highlight the wisdom inherent in PrEP mistrust as a reflection of trans women’s experiences that underscore the broken bonds of trust between communities, researchers and the research enterprise. PrEP mistrust is amplified through perceived paradoxes that suggest to trans women that they are key experimental participants but not target PrEP users outside of research settings. Findings highlight the urgent need to reframe mistrust not as a characteristic of trans women to be addressed through education and outreach, but as a systemic institutional‐ and industry‐level problem replicated, manifested and ultimately to be corrected, through global HIV science.

## 1 Introduction

Pre‐exposure prophylaxis (PrEP) is a leading strategy to prevent HIV. Yet, with the exception of white gay men residing in the United States and high‐income settings in the Global North, PrEP uptake remains suboptimal worldwide [1]. In Latin America, Brazil has been leading PrEP implementation offering PrEP at no cost, since 2017, through the Brazilian Public Health System (SUS) to populations classified at substantial risk for HIV infection [2]. Nonetheless, PrEP awareness and uptake remain low [3, 4]. Obstacles globally include cost, challenges with health system integration and political will. Emerging scholarship has also highlighted mistrust as a critical barrier to PrEP acceptability [5, 6, 7]. Understanding mistrust of biomedical HIV prevention is particularly important as PrEP constitutes a paradigm shift from condom‐based prevention, which requires significant trust in the systems that promote the use of such biomedical interventions for people who are not living with HIV [8].

Medical mistrust is a complex global phenomenon referring to the lack of trust *and* associated suspicions of the medical system, including sentiments such as, not trusting the medical system to treat you appropriately as a patient, not trusting the motives of those involved in the medical system (particularly clinical researchers), not believing that the system will prioritize your needs and care [9, 10]. Increasingly, scholarship is shifting from assessing mistrust as an isolated characteristic of certain populations (e.g. African American’s mistrust of biomedical research in the United States) to evidencing its genesis in pervasive and institutionalized oppressive injustices exercised against marginalized communities in clinical settings [11, 12, 13]. Indeed, medical care and research exist within and contribute to systems that create, sustain and reinforce racism, homophobia, transphobia, classism and other stigmas, suggesting that people’s experience of these systemic injustices often underpin medical mistrust [14]. It is only by recognizing medical mistrust as systemically produced and reproduced that structural solutions can be meaningfully addressed by researchers and clinicians.

Present‐day PrEP research contributes to and interacts with a problematic legacy of HIV experimentation on marginalized communities in resource‐limited settings [15]. Instances of community rejection of early PrEP trials underscore the problematic power differentials in North–South collaborations that can perpetuate breaches in researcher‐community relationships and lead to the rejection of offshore research [16]. More recently, PrEP has spurred the “projectification” of research, referring to the seemingly endless series of isolated, short‐term trials that are not subsequently integrated into existing healthcare systems, even when interventions are proven to be efficacious [17, 18]. Within this context, “key populations,” [19] classified as at highest risk for HIV and thus with the highest need for prevention, are of particularly high value to prevention investigators. Often marginalized by society at large, these populations have been central to PrEP trials, demonstration projects, and open‐label studies exploring how PrEP works in “real world” settings. Because marginalized “key populations” have played an indispensable role in advancing PrEP knowledge, a deeper understanding of how mistrust is reproduced among them within the context of PrEP science is urgently needed.

Peru is a particularly relevant setting in which to assess how mistrust perpetuated by HIV science shapes community acceptability and uptake of PrEP. Peruvian men who have sex with men (MSM) and trans women constituted more than half of the sample for the watershed iPrEx trial, which demonstrated the efficacy of PrEP for HIV prevention [20, 21]. In the aftermath of iPrEx and numerous PrEP implementation challenges that followed, emerging research among Peruvian MSM and trans women has highlighted the existence of mistrust of international drug trials, foreign investigators and Peruvian institutional actors implementing the studies as obstacles to widespread PrEP acceptance and use [22]. Notably, PrEP was approved by the Peruvian FDA in 2017 and yet, there is no current integration within Peru’s public health system and availability for trans women occurs primarily in the context of HIV biomedical prevention research [23, 24]. In this paper, we used qualitative methodology to explore the mistrust and its impact on research participation as reported by trans women who participated in a PrEP adherence trial and those who refused involvement in the study (i.e. voluntarily withdrew, and/or were lost to follow‐up).

## 2 Methods

### 2.1 Study design

Data are derived from a social network‐based PrEP adherence intervention for trans women in Lima, Peru (TransPrEP, R34 MH105272; clinicaltrials.gov #NCT02710032). TransPrEP was a cluster‐randomized trial with two arms and six cohorts from three areas of Lima. Participants received 6‐months of PrEP, routine HIV/STI testing, and two counselling sessions; those randomized to the intervention were offered 14 community‐based adherence workshops. The full study methods have been published previously [25].

Specifically, this paper draws on focus groups (FGs) and interviews with 86 trans women who participated in the formative and/or evaluative stages of TransPrEP. Eligibility criteria included being aged 18+, assigned male sex at birth and identifying on the transfeminine continuum (e.g. “trans,” “transgender,” “travesti”). Purposive sampling [26] was implemented in collaboration with a trans community centre, *Femínas*, for recruitment across formative and evaluative stages with participants who consented to participate.

The formative stage (May‐July 2015) consisted of 4 focus groups (FGs; n = 32), 20 interviews (16 community interviews and 4 community leader interviews) and brief demographic surveys. The primary aim of the formative work was to inform the design and acceptability of the forthcoming PrEP adherence intervention. Key domains within the semi‐structured guides for FGs and in‐depth interviews included: HIV prevention strategies, HIV vulnerabilities, social network structures, acceptability of and preferences for different HIV prevention methods, and awareness and hypothetical acceptability of PrEP for HIV prevention. The evaluative stage (April‐May 2018) involved 34 interviews with trans women who had been recruited and deemed eligible for the trial. The evaluation aimed to explore strengths and weaknesses of study participation, as well as drivers of non‐participation. Participants at the evaluative stage included: peer intervention workshop facilitators (n = 4); participants who screened but never enrolled (n = 8); participants who enrolled in the study but later dropped out (n = 6); and intervention completers (n = 16). The semi‐structured interview guide probed on the following: recruitment, enrolment, counselling session, retention efforts, workshops (if randomized), experience with PrEP, motivators for continued participation, rationale for study discontinuation and research mistrust.

Institutional review boards of *Asociación Civil Impacta Salud y Educación* (IRB #0089‐2014‐CE) and the University of California, Los Angeles (IRB #13‐001898) approved this study. All participants provided written consent before beginning any study procedures and were provided 15 *Nuevos Soles* (approximately $5 USD) for their participation.

### 2.2 Analytic approach

FGs and interviews were conducted in Spanish by native speakers who were part of the research team, audio recorded and transcribed verbatim. Immersion crystallization [27] guided the analytic approach which entailed a close read of the raw data alongside semi‐structured guides codebook creation and line‐by‐line review of the transcripts to attribute codes. This iterative approach emphasizes examining data and reflecting on the experience of being immersed in the data analysis at three‐time points; formative analysis, evaluative analysis and analytic comparison across stages. Data collection was conducted across two time points, and the initial formative work in 2015 was not designed to probe questions related to mistrust, but rather, general acceptability, interest, and potential barriers to implementing a PrEP intervention. Nevertheless, mistrust emerged as a key participant concern in the first iteration of the codebook. In 2018, having identified this theme, question guides were specifically developed to query further on PrEP and research mistrust. Transcripts were analysed based on an inductive and deductive approach to identify codes and relationships between codes in the iterative development of the codebook. The same two independent reviewers (APB and SNM) participated in codebook creation and systematic coding across the multiple qualitative data sources. The analytic approach was triangulated across FGs and interviews though code comparison and also through the creation of memos to expand upon coded text and describe relationships between the codes [28, 29]. Any coding differences encountered were discussed and reconciled at regular team meetings. Analyses were conducted using Dedoose (2014, Los Angeles, CA, USA: SocioCultural Research Consultants, LLC) and all reported data utilizes pseudonyms to protect participants’ identity.

## 3 Results

Three paradoxes of trans women’s involvement in PrEP science as members of a “key population” emerged: The first underscores the perceived discordance between the increased frequency of PrEP research targeting trans women amid limited improvements in HIV outcomes among trans women. The second illustrates the continued dismissal of participant reports of PrEP‐related side effects by research staff, despite their status as a “key population.” The third highlights the contradiction that PrEP is tested on trans women but is not readily available to them, fostering feelings of the community’s use as a “guinea pig” to advance PrEP science.

In the formative stage, 32 trans women participated in four focus groups. The mean age for focus group participants was 29 years and the majority (75%) reported completing secondary school. Twenty interviews were also conducted in the formative stage across 16 community member interviews and four community leader interviews with a mean age of 30 years. The evaluation stage (mean age 28 years) consisted of 34 interviews with four peer facilitators, eight participants who screened but never enrolled, six participants who enrolled in the study but later dropped out, and 16 intervention completers. Sex work was the most reported occupation across all stages: 41% of focus group participants, 40% of interview participants and 47% of evaluation participants. Following sex work, most prevalent occupations reported were cosmetician/hairdresser, study coordinator or peer health promoter.

### 3.1 Paradox 1: why more research, but not more health?

All 86 trans women who participated in this study had prior experience as a research participant or knew a close acquaintance that had participated in HIV prevention research (e.g. antibody, vaccine, or PrEP trials). Participants were highly knowledgeable about the HIV research landscape, including the distinct incentives and treatments offered across current and prior studies. iPrEx was consistently named as the beginning of a wave of HIV prevention studies targeting trans women; “iPrEx was just the start and many more studies have followed” (Facilitator; Ruty 34 years old).

The large volume of new HIV prevention studies targeting trans women was described as fostering a competitive recruitment climate. A study facilitator (i.e. staff member) described fielding questions about the different studies noting, “it is hard to know how to respond since often they know more about the studies from friends who are participating than I do” (Angelica 24 years old). The choice to participate in an HIV study is also complicated by limited HIV prevention and care options for trans women in the existing healthcare system. Studies were often described as providing first‐line HIV treatment and prevention services; “I get my [medical] care through these studies” (Active participant; Lorena, 27 years old). Another participant stated, “I found out about this study because I needed to get an HIV test and a friend told me that the easiest way to get a free test is through a study. I did, then I learned about PrEP and wanted to participate” (Active participant, Luisa 19 years old).

However, even those who noted the importance of HIV research for access to care were wary of the surge of clinical trials and noted that the health impact for trans women in Lima was limited: “there are more and more studies, but we still get sick” (Facilitator; Nena, 28 years old). Moreover, care was described as ephemeral and HIV acquisition frequently posited as inevitable: “I participate in studies now so that I can get care. But what happens once I get sick? These studies end” (Active participant, Danica 27 years old). Andrea shared, “just like my friends, I was always being contacted to participate in studies, until I found out I was positive. Since then, no research is interested in me” (Formative interview, Andrea 36 years old).

While many participants said they valued the care provided by studies, they questioned the lack of long‐term health benefits for trans women, and this was further complicated by the wariness of foreign investigators perceived to be driving the PrEP research agenda. For example, Maria noted “how long have gringos been researching us? Why are we still getting sick? What will I do if I get HIV?” (Screened but never enrolled; Maria 35 years old). Similarly, Ruty discussed cautionary tales circulating among the trans community about PrEP:

“People talk and trans women know of iPrEx and other American studies. They often tell me that they don’t believe in the pills, because they have heard of people that participated and now have HIV… so many of us are sick that it raises doubts that the research helps.” (Facilitator; Ruty 34 years old)

Several active participants expressed fear that their access to PrEP would end with the current study, even though all participants who wanted to continue PrEP were offered a free temporary transition plan. As described, “I wish I could afford to pay for PrEP and continue to take this medicine” (Active participant; Rocio, 19 years old), another when asked about plans after the study’s end noted “I would like to continue PrEP, do you know of other studies that are offering it?” (Active participant; Jahaira, 24 years old). While all participants were offered a transition plan to ensure continued temporary PrEP access after the study ended, a peer facilitator commented, “I’ve told them about other studies, they tell each other about the resources they learn of, but when will they be actually able to afford to take PrEP outside of research?” (Facilitator; Ruty 34 years old).

### 3.2 Paradox 2: who is PrEP really for?

The second paradox identified centres on transphobic mistreatment of participants and dismissal of PrEP‐related side effects by research physicians and staff, even in studies designed to ensure PrEP is attuned to trans women’s needs. One FG participant voiced “they (study staff) don’t care about us; they just want us to participate… They ignored my question if it [PrEP] would affect my hormones” (Formative FG #1). In response, another participant commented “Yeah, trans women have different needs and doctors and researchers don’t listen to us… we take hormones, we drink, and adding so many pills isn’t good for us” (Formative FG #1). Another participant who enrolled and dropped out stated, “I was nervous about taking PrEP with my hormones. When I asked the nurse, she just made me feel bad for taking hormones” (Enrolled and dropped out; Gaby, 24 years old). Indeed, lack of information on potential interactions with hormone therapy emerged as a key barrier to taking PrEP.

Participants taking PrEP also described being dismissed when telling research staff about side effects. Maya, who enrolled and dropped out of the study, shared, “side effects were the reason I stopped taking the pills. I told the doctor, but he didn’t listen… he just said to keep taking them and wait two weeks” (Enrolled and dropped out; Maya 25 years old). Furthermore, Michi described:

“I didn’t want to keep going to the clinic. I know I said I would as part of the study, but PrEP made my head hurt and that one doctor didn’t believe me and he kept making me feel bad…they were obviously only giving me medical attention because they had to” (Enrolled and dropped out; Michi, 25 years old).

Whether experienced first‐hand or shared among friends or acquaintances, PrEP side effects were described as particularly relevant given the social realities of trans women, such as engagement in sex work:

“My friend who was part of the first PrEP study told me that she had horrible diarrhea and stopped taking the pills so she couldn’t work [as a sex worker]… Because she is trans, the doctors didn’t believe her, and I know of many other trans women that dealt with stomach issues and couldn’t take the medicine anymore. They needed to work so they could eat.” (Enrolled and dropped out; Nanci, 26 years old).

Repeated negative interactions with medical providers during study visits, and in clinical spaces designed to support PrEP research studies, amplified the perception of ill will towards trans women and aligned this perception with their understanding of clinical trials. As forcefully articulated, “they don’t listen to me when I say PrEP makes me sick. Would they listen to a man if he said PrEP made him sick?” (Enrolled and dropped out; Ana, 29 years old). This disregard of basic concerns by investigators and staff fosters suspicions that prioritizing trans women as a “key population” is disingenuous and calls into question who PrEP is really for.

### 3.3 Paradox 3: are we just guinea pigs?

The third paradox highlights the contradiction that PrEP has been tested repeatedly on trans women but is not readily available to them, furthering the belief that their community is being used as “guinea pigs” rather than as PrEP’s intended users. PrEP mistrust was further amplified by the conflict between the short‐term rewards, provided by financial incentives and temporary access to new medications, against long‐term public health failures, leading to limited access for successful therapies and care. As described by Angelica:

“What can I tell you? Do I have a problem that my community is treated like an experiment? Yes. But we continue to participate and work for these studies because we are seen… we at least get some HIV care, sometimes we get PrEP, and we even get paid” (Facilitator; Angelica, 27 years old).

Several trans women discussed how the majority of PrEP research conducted in Peru primarily benefits the Global North and does not address the real health needs of their community. Participants voiced suspicion of foreign funding for PrEP research that emphasizes trans women as a “key population,” noting that it felt disingenuous. For example, “we are experiments for other countries to see whether or not we react well to the pills” (Enrolled and dropped out; Dani, 22 years old). As one FG participant queried, “Why are we [trans women] being used like guinea pigs, every single one of us?” (Formative FG #1). Narratives also questioned the classification of trans women as “high‐risk” (especially compared to the cisgender male sexual partners who are their main source of HIV exposure). One participant stated “it would be ideal to give this [PrEP] to married men. They are the ones that ask us not to use a condom.” (Formative FG #2).

Further contributing to the perception that trans women were simply “guinea pigs” were frustrations reported over the physical burden of repeated behavioural and biological measures and studies’ emphasis on obtaining diverse clinical samples. As stated, “everyone knows, first you do a long survey, if not multiple… then they take your blood…” (Active participant; Carla, 23 years old). Another participant elaborated that the request for a hair sample in addition to a blood test, specifically, made her feel more vulnerable to experimentation: “I didn’t want to [give them a hair sample], I told them no… I felt strange because it was my hair, from then on I felt like they were experimenting on me, I won’t lie to you” (Enrolled and dropped out; Dani, 22 years old). Extraction of blood and hair highlighted the perceived commodification of trans women’s bodies; “people think that all they [study staff] want is your blood… all these studies all they want is blood…and friends have warned me that gringos want to take your blood because later they will sell it.” (Active participant; Fiona, 31 years old). One FG participant noted “I won’t participate in another study that takes my blood. We don’t trust what they do with our blood” (Formative FG #2). These sentiments highlighting suspicions about and motivation for biospecimens collect were commonly echoed by participants across all stages of data collection.

## 4 Discussion

### 4.1 PrEP Mistrust and the dilemmas of being a ‘key population’

To understand the experiences and perceptions of trans women who interacted with a PrEP intervention, we interviewed 86 trans women across two timepoints. What initially began as an attempt to improve our own study design resulted in revealing a critical tension relevant to the broader project of HIV prevention globally. Findings suggest an urgent need to better understand mistrust, not as a trait of trans women addressed solely through education about the research process, but as a justified response to systemic inequities replicated by and manifested through global PrEP research. Narratives highlight the wisdom inherent in PrEP mistrust as a reflection of learned community insights that underscore the broken bonds of trust between communities, researchers and the research enterprise. PrEP mistrust is amplified through perceived paradoxes that suggest to trans women that they are key experimental participants but not target PrEP users outside of research settings.

Community‐level recognition of the sheer volume of HIV prevention studies targeting trans women underscores the discordance between increases in PrEP research targeting trans women and limited improvements in HIV outcomes in the same population. Aligning with research from the United States [30]. our findings demonstrate how this tension fosters research mistrust at different points across the research continuum; from formative research to trial enrolment, refusal and loss to follow‐up. Trans women not yet enrolled and those who withdrew from the study reported a particularly high awareness of the legacy of iPrEx in addition to the numerous subsequent HIV biobehavioural studies funded primarily by the United States. Participants were acutely aware of research dynamics whereby Global North researchers bring funding, drive project priorities, and reap the benefits of study findings as PrEP remains unavailable in‐country outside of research contexts. Furthermore, within this context, trans women described deep knowledge of the potential benefits (i.e. first‐line therapies, financial incentives) and extractive elements of these studies (e.g. collection of biological samples, time commitments, etc.). Importantly, the decision to participant (or not) was based not on the absence of mistrust, but on weighing the potential benefits presented by the study against the concurrent risks of participation.

Narratives noted the ways in which trans women perceived themselves as conveniently “key” to experimentation, bringing forth concerns regarding who PrEP is really for and the dire gaps in HIV prevention access for trans women in the Global South. The frequent mistreatment experienced by participants translated into cautionary tales within the community, transforming individual level experiences into widely circulated warnings that impede future participation in PrEP research. Indeed, actual and perceived experiences of transphobic mistreatment included interpersonal interactions with research clinicians (dismissal of side effects, failure to disclose needed information) and subverted beneficence for participants (scientific knowledge production that benefits only the Global North). Recognizing the wisdom of PrEP mistrust underscores that trans women experience a range of discomforts and, at times, maltreatment within PrEP science. Our findings contribute to ongoing critical global health dialogues by advancing the understanding of medical mistrust as not simply the absence of trust but rather a dynamic involving increased suspicions around the medical system and the motivations/benefits of the PrEP research industry.

A fundamental challenge in HIV prevention efforts is the delicate balance between identifying trans women as a “key population” benefitting from HIV research, and the burden of investigative practices that leave trans women feeling like guinea pigs. This finding echoes calls to address emotional trauma emergent from research fatigue [31] and to end stigmatizing language in prevention science (i.e. use of the term “high‐risk”) that obscure the pervasive social inequities that increase vulnerability to HIV [32, 33]. Our findings support literature emphasizing the importance of meaningful community engagement at all stages in the research process as a strategy to improve procedural ethics, quality, acceptability and sustainability of research [34, 35, 36]. However, these actions are not enough. There is a need to incorporate justice paradigms [37], including insights from intersectional feminism and critical race theory [9, 38, 39, 40], within HIV prevention science to account for the dynamics of power and exploitation that foster inequities embedded within research and better engage with community values on what it means for science to be trustworthy.

Several limitations of this study should be considered. While these qualitative findings are derived from a large sample across diverse stages of the research continuum, all participants lived in Lima, Peru and thus results may not be generalizable to other trans women in Peru or beyond. Perceptions of the rationale for and conduct of biomedical HIV prevention research were limited to trans women who were willing to participate in at least the formative component of the TransPrEP study. While we interviewed trans women who discontinued participation in the study after enrolment, future research on mistrust would benefit from speaking to trans women who refused any participation.

## 5 Conclusions

The participation of trans women is crucial to advance PrEP science and ensure that PrEP, as a novel prevention tool, is attuned to their needs. Indeed, modelling studies suggest that PrEP, offered alongside sexual health promotion strategies (i.e. condoms), may avert 50% to 60% of new HIV infections among Peruvian trans women who engage in sex work over the next decade, if it is used at sufficient levels [41]. Given the potential impact of PrEP scale‐up there is additional responsibility by researchers to actively promote strategies to build and sustain equitable relationships in PrEP research. Yet, narratives from Peruvian trans women who have participated for years across numerous PrEP studies challenge the positioning of trans women as “key” to PrEP rollout, given that they are rarely treated as such outside of study environments.

These accounts highlight how trans women’s concerns about PrEP are frequently dismissed and how the increasing burden of PrEP research heightens sentiments of being a key test subject but not a key beneficiary. Our findings show how the perceived and actual harms of experimentation are compounded by the problematic reality that there are no long‐term strategies to support PrEP access for trans women once research ends. Therefore, trans women do not meaningfully benefit from the research they are central to. In recognizing the contradictions inherent in being classified as a “key population,” PrEP mistrust is both pervasive and justified. This paper should serve as a call to action to reckon with PrEP mistrust as a systemic institutional‐ and industry‐level problem that is replicated and manifested through global HIV prevention research.

## Competing interests

No potential competing interest was reported by the authors.

## Authors’ contributions

APB, JLC, SLR, JRL, JS and KHM conceptualized and designed the study. APB, LH, AS, XS and SNM supporting in data collecting. APB, SNM and JLC conducted data analysis and drafted manuscript. All authors reviewed manuscript and supported with edits.

## Data Availability

The data that support the findings of this study are available from the corresponding author, APB, upon reasonable request.
